# Artificial intelligence for dental caries diagnosis: translating algorithms to clinical practice

**DOI:** 10.3389/fmed.2026.1879708

**Published:** 2026-07-29

**Authors:** Ziyu Wang, Zixuan Zhu, Ti Jiang, Yuanping Gu

**Affiliations:** 1 School of Stomatology, Guilin Medical University, Guilin, China; 2 Clinical Medical College, Guilin Medical University, Guilin, China

**Keywords:** artificial intelligence, clinical decision support, deep learning, dental caries diagnosis, domain shift, explainable AI, multicenter datasets

## Abstract

Dental caries remains the most prevalent chronic disease worldwide, affecting more than two billion people and driving substantial healthcare costs. While early detection is central to minimally invasive dentistry, traditional diagnostic methods suffer from limited sensitivity and high inter-examiner variability, especially for incipient lesions. Artificial Intelligence (AI) has shown promise in overcoming these limitations, and many studies have reported expert-level performance under controlled conditions. However, a substantial translational gap persists between algorithmic success in silico and reliable performance in real-world clinical environments. This review synthesizes the full development pipeline of AI for caries diagnosis—from data curation and ground-truth construction to model design, validation, and clinical deployment. We highlight persistent bottlenecks including domain shift across imaging devices and clinical settings, subjective and inconsistent annotation practices, limited multimodal datasets, and heterogeneous reporting standards. Emerging strategies such as multi-center data collection, probabilistic labeling, self-supervised learning, domain adaptation, and test-time augmentation offer partial solutions but remain underutilized. We argue for a paradigm shift from binary detection toward quantitative, risk-based staging that aligns with minimally invasive dentistry and the WHO Global Oral Health Action Plan 2023–2030. By advocating for standardized multimodal datasets, rigorous external validation, explainable interfaces, and human-centered clinical integration, this review outlines a roadmap for translating AI innovation into trustworthy, equitable, and clinically meaningful decision-support systems capable of reducing the global burden of untreated caries.

## 1 Introduction

Dental caries is a chronic, multifactorial disease characterized by the demineralization of tooth hard tissues (1). Untreated caries in permanent teeth affects billions of people worldwide, imposing disproportionate costs on low- and middle-income populations (2). The World Health Organization’s Global Oral Health Action Plan 2023–2030 ambitiously aims to reduce the prevalence of untreated dental caries by 25% by 2030 (3).

Visual-tactile examination and two-dimensional radiography in traditional diagnostics are limited in detecting early demineralization (4). Poor inter-examiner agreement, particularly for incipient lesions, leads to inconsistent treatment decisions. While adjunct technologies like laser fluorescence and near-infrared transillumination improve sensitivity (5, 6), they are costly and have variable performance. Artificial intelligence (AI), particularly convolutional neural networks (CNNs), has emerged as a powerful tool for automated image analysis (7). In dental caries, detection, segmentation, classification, and risk prediction have all benefited from the applications of AI to various imaging modalities. In controlled studies, CNN classification and semantic segmentation models (e.g., ResNet-50, U-Net) demonstrate high diagnostic performance, achieving area under the receiver operating characteristic curve (AUC) metrics of 0.76–0.98 that match or exceed expert performance (8–14). However, under external validation across different datasets, these metrics routinely degrade to AUC values of 0.53–0.57 due to severe domain shift (13, 15).

In this work, we examine the entire AI development pipeline, analyze the translational gap between algorithmic performance in silico and clinical utility at the chairside, and highlight persistent obstacles such as domain shift, annotation scarcity, and the lack of standardized reporting. We identified relevant literature through structured searches in PubMed and IEEE Xplore by combining AI concepts, caries concepts, and modality/context. We synthesize a comprehensive framework for developing robust, clinically deployable AI systems for caries diagnosis from three interconnected yet distinct angles: data, algorithms, and clinical translation. Each section integrates what has been done to date and what should follow next.

Given the rapidly evolving nature of AI applications in dentistry and the broad scope of methodologies involved, this article is designed as a narrative review that integrates a comprehensive synthesis of the published evidence with forward-looking expert perspectives. Unlike systematic reviews, we did not perform a formal risk-of-bias assessment or meta-analysis. Instead, we adopted a structured literature search strategy (detailed in Section 2) to identify key studies across the development pipeline—from data curation to clinical deployment. Based on this evidence base, we supplemented our findings with critical appraisal and reasoned opinions to identify persistent gaps and propose actionable solutions. We explicitly frame our discussion around three interconnected pillars—data, algorithms, and clinical translation—to bridge the gap between current engineering achievements and real-world clinical needs. This hybrid approach allows us to not only summarize what has been accomplished but also to advocate for a paradigm shift in how AI systems for caries diagnosis should be developed, validated, and integrated into practice.

## 2 Methodology

Given the narrative nature of this review, we did not perform a formal systematic meta-analysis but conducted a comprehensive, structured literature search to ensure broad coverage of the topic.

### 2.1 Databases searched

The primary electronic databases searched were PubMed and IEEE Xplore, supplemented by Google Scholar to capture interdisciplinary studies and recently published online-first articles not yet fully indexed in MEDLINE.

### 2.2 Time period covered

The search period covered publications from January 2020 to October 2025. The starting year was selected to coincide with the widespread adoption of deep learning techniques in medical image analysis, ensuring the inclusion of foundational as well as state-of-the-art studies.

### 2.3 Search strategy

The search strategies on PubMed and IEEE Xplore combined Medical Subject Headings (MeSH) and free-text keywords, structured as follows:(dental caries or tooth decay) and (ai or deep learning for cnn) and (radiograph or bitewing or panoramic or cbct or x-ray or intraoral or periapical radiograph). For Google Scholar, we used analogous keyword combinations with broader filters to identify supplementary literature, grey literature, and preprints; the first 200 records sorted by relevance were manually screened for potential inclusion.

### 2.4 Criteria for article selection

For article selection, we prioritized peer-reviewed original research, systematic reviews, and clinical trials published in English. We excluded conference abstracts and studies not specifically addressing image-based (radiographic, photographic, or optical) caries detection. When multiple studies from the same cohort were identified, we retained the most comprehensive or recent report. Additionally, the reference lists of all included articles and existing systematic reviews were manually cross-checked to identify any relevant studies missed by the initial database queries. The final inclusion of studies was iteratively discussed and agreed upon by all authors to align with the thematic framework of this review.

## 3 Data: the foundation and bottleneck

### 3.1 The pervasive challenge of domain shift

Domain shift, i.e., the degradation of model performance when applied to data different from the training set, is particularly acute in dental imaging. Deep learning caries detectors exhibiting superior performance on specific training sets may struggle in heterogeneous clinical environments, when images are acquired with different X-ray machines, sensors, formats, or annotation styles (16, 17). This problem arises from:

- Imaging equipment: Differences between sensor types (CCD vs. CMOS), manufacturers, and imaging protocols (kVp, mA, exposure time) create systematic shifts in image appearance that models learn as spurious correlates.
- Patient demographics: Anatomical variations across age, ethnicity, and populations alter the distribution of tooth morphology and radiographic contrast.
- Clinical conditions: Variations in saliva, presence of restorations, and disease prevalence change the background and signal-to-noise characteristics of images.

Comparative studies have shown measurable drops in AUC and sensitivity when models trained on one vendor’s images are tested on another’s (17, 18). Several recent studies have begun to address generalizability through either external validation or limited multi-institutional data collection, although the comprehensive evaluation of cross-device domain shift remains uncommon (19–22). Others have applied domain adaptation techniques (e.g., adversarial domain adaptation, feature alignment) or test-time augmentation to improve performance (23–25). While unsupervised domain adaptation and self-supervised pretraining recover 0.1–34.2% of the precision drop on out-of-distribution datasets, hardware-specific high-frequency noise limits such recovery (26–28). Similarly, local contrast-enhancement strategies (e.g., CLAHE) improve early enamel lesion sensitivity but amplify radiographic noise, substantially increasing false-positive rates (29).

To achieve cross-scenario robustness, future work must construct multi-center, cross-device standardized datasets and optimize Transfer Learning and Domain Adaptation algorithms. During training, data should be intentionally collected from diverse sites and devices, and models should be evaluated on held-out centers. Establishing representative global oral imaging databases can systematically reduce the negative impact of hardware variability on diagnostic accuracy.

### 3.2 The ground truth paradox and annotation scarcity

The “ground truth” in curated datasets suffers from inherent subjectivity. In most clinical imaging studies, histological gold standards like extracted tooth tissue sections are difficult to acquire at scale, and annotation consensus among multiple clinicians is often considered the reference standard. However, this creates the primary bottleneck in clinical validation (30, 31).

Pairwise comparisons of expert annotations reveal low overlap (e.g., IoU values reported in the literature) (17). Probabilistic annotation strategies (e.g., Gaussian Mixture Models to convert multiple annotations into continuous label heatmaps) have been proposed and piloted to preserve inter-expert uncertainty rather than collapsing it into a single binary mask (32). Self-supervised learning (SSL) approaches (e.g., SimCLR-style pretraining) have been applied to unlabeled CBCT and radiographic data to reduce dependence on costly annotations, and early results show measurable gains in downstream classification/segmentation performance (27, 33).

The field should scale probabilistic annotation pipelines and integrate SSL pretraining into standard workflows. Specifically, multi-expert probabilistic labels should be used as training targets (rather than hard consensus masks), and SSL pretraining on large unlabeled clinical image corpora should be adopted as a routine step before supervised fine-tuning. Meanwhile, research consortia should pilot the collection of biologically meaningful benchmarks (e.g., micro-CT, polarized light imaging, and other ex vivo modalities) on subsets of cases to provide objective anchors for algorithmic calibration. These biological benchmarks are not yet widely available, but targeted efforts (e.g., in academic centers with access to extracted teeth) can create seed datasets that improve model interpretability and validity.

### 3.3 Toward multimodal and standardized datasets

The definition of next-generation scenarios must evolve toward “whole-pipeline decision support” to bridge the gap between engineering R&D and clinical management. The future lies in curated, multimodal public datasets that balance standardization with robustness.

Some datasets now pair radiographic images with clinical metadata. For example, a few pilot datasets (e.g., MMDental and other institutional collections) combine CBCT with annotated clinical records and limited longitudinal follow-up (34, 35). High-resolution modalities like intraoral optical coherence tomography (OCT) are also being integrated into research datasets to provide richer features for early lesion detection (36, 37). This integration facilitates a leap from simple qualitative detection to quantitative staging and longitudinal risk assessment. For example, quantitative staging is feasible by calculating the demineralization-depth-to-enamel-thickness ratio from pixel segmentations and mapping directly to ADA CCS categories: initial (enamel-only; indicating non-invasive remineralization), moderate (outer dentin; indicating micro-invasive sealants), and advanced (inner dentin; requiring minimally invasive restoration) (38).

In addition to constructing multi-center, cross-device standardized datasets, the mitigation of domain shift involves the following:

- Expanding datasets to include multimodal inputs: intraoral photographs, bitewing and panoramic radiographs, OCT, and where feasible, non-imaging data (patient history, caries risk factors, fluoride exposure, microbiome/salivary biomarkers).
- Collecting longitudinal data and training models for lesion activity assessment and progression prediction.
- Standardizing annotation protocols and metadata schemas to ensure interoperability.

## 4 Algorithms: toward precision in prediction

### 4.1 Dominant architectures and tasks

Most AI applications in caries diagnosis utilize supervised learning, where models are trained on expertly labeled datasets to execute specific clinical tasks. The three dominant task paradigms in the current landscape, i.e., classification, object detection, and segmentation, form a hierarchy of clinical relevance based on their spatial localization capabilities and diagnostic depth (39).

- Classification (ResNet-style): widely used for rapid triage; many studies report high accuracy on curated datasets (40–44).
- Object detection (YOLO family, EfficientDet, RetinaNet): used for multi-tooth localization and multi-category lesion screening; single-stage detectors have been optimized for speed and are being adapted for edge devices (45–50).
- Segmentation (U-Net family, nnU-Net): provides pixel-level masks for volumetric quantification and ICDAS mapping; nnU-Net and self-configuring frameworks have set performance benchmarks in segmentation tasks (51–58).

These architectures have complementary strengths. For example, YOLOv5 has demonstrated significantly lower false negative rates compared to EfficientDet in certain tasks (32), but YOLO underperforms other models in scenarios requiring high precision on small or occluded objects or complex scenes (59–61). Some experimental evidence suggests that ensemble strategies can significantly improve performance metrics related to false negatives (sensitivity/recall) compared to single models (11, 40, 62–64). Table 1 summarizes representative studies highlighting diverse architectures, modalities, and performance benchmarks in the field.

**Table 1:** Representative studies with different architectures, modalities, and performance benchmarks.

Author	Date	Modality	Task	Architecture	Key performance/metric	Main limitations
Karakuş (48)	2024	Bitewing radiographs	Detection	YOLOv8	Precision: 97.7%, Sensitivity: 93.2%, F1: 95.4%	Performance drops on panoramic images; struggled with buccal caries
Lian (40)	2021	Panoramic/periapical	Segmentation and classification	Cascaded (nnU-Net + DenseNet121)	IoU: 0.785, Accuracy: 98.6%, D1 Accuracy: 95.7%	High computational cost of cascaded stages; single-center dataset
Felsch (65)	2023	Intraoral photographs	Classification	SegFormer-B5 (Vision Transformer)	Accuracy: 97.8%, IoU: 0.959	High dependence on image quality/lighting; needs external validation
Mertens (85)	2021	Bitewing radiographs	Detection (RCT)	FCNN (ResNet-style)	Sensitivity: 0.81 (AI-support) vs. 0.72 (Dentists alone)	Risk of over-treatment for incipient lesions; subjective ground truth
Balachandran (78)	2026	Intraoral imaging	Classification	EnamelNet-TRiX	Accuracy: 99.25% (Internal), 96.33% (External)	Limited data, poor borderline discrimination and practical deployment drawbacks
Lukáš Kunt (16)	2023	Bitewing radiographs	Detection	Ensemble 3: RetinaNet-SwinT, Faster R-CNN- ResNet50, YOLOv5-M, and RetinaNet-R101	Precision:0.83, Recall:0.77 F1:0.80	Two drawbacks: inconsistent image scaling and single annotator labeling
Zanini (27)	2024	CBCT	Classification	ResNet-18	F1: 88.42% Precision:90.44%	Dataset limitations: only single-cavity images from one CBCT scanner

Future directions for architectural development include:

- Transitioning from binary caries/non-caries classification toward fine-grained, ordinal architectures capable of mapping pixel-wise segmentation masks to clinical staging systems like ICDAS or ADA CCS.
- Developing hybrid attention-based architectures [e.g., Vision Transformers (65)] that integrate local feature extraction with global contextual awareness to differentiate between anatomical variants and actual pathology.
- Enhancing the algorithmic granularity of detection tasks by moving from coarse bounding boxes to instance segmentation, allowing for the precise measurement of caries-to-tooth volumetric ratios.

### 4.2 Multimodal/integrative diagnostic approaches

Pioneering research is moving beyond isolated image analysis toward multimodal and integrative approaches that reflect the biological complexity of caries as a biofilm-mediated disease. High-resolution technologies such as intraoral OCT and multimodal datasets that pair radiographs with visible-light images are being used in proof-of-concept studies to improve the detection of incipient lesions (36, 66–68). Fusion strategies (early fusion, late fusion, attention-based fusion) are also explored to combine complementary modalities (69–72).

Future systems must evolve from image classifiers to integrated diagnostic assistants, which involves:

- Fusing imaging data from multiple sources (e.g., combining intraoral photographs with bitewing radiographs for a more complete assessment).
- Incorporating non-imaging data such as patient history, caries risk factors (diet, hygiene, fluoride exposure), and microbiome/salivary biomarkers.

### 4.3 Addressing resource constraints and real-world deployment

The clinical deployment of AI demands a dual achievement of real-time, second-level feedback and fine-grained differentiation (e.g., enamel vs. dentin caries). To meet these conflicting demands of precision and speed, algorithmic evolution has branched into two distinct specializations based on the clinical environment. Lightweight one-stage detectors (YOLO family and derivatives) have been optimized for mobile and edge deployment, and sub-100 ms real-time inference seems feasible with current hardware and optimization methods (73–75). In contrast, complex two-stage and ensemble pipelines have demonstrated superior performance in difficult imaging scenarios (e.g., secondary caries near restorations) but require advanced computation hardware (e.g., RTX 3090-class GPUs) for training and inference (11, 21, 76).

Future research needs to:

- Continue to develop hybrid edge-cloud architectures, with lightweight edge models for immediate screening and cloud-based high-precision models for complex cases (77, 78). This edge-cloud-clinic closed-loop should be coupled with continual learning mechanisms that incorporate clinician feedback and local data drift monitoring (79, 80).
- Invest in model compression (81), knowledge distillation (82), and self-adapting segmentation frameworks (83) (e.g., lightweight nnU-Net variants) to bring high segmentation precision into resource-constrained settings.
- Validate mobile deployments in field studies that measure not only diagnostic metrics but also workflow integration, clinician acceptance, and downstream treatment decisions (84).

## 5 Clinical implementation: from metrics to medicine

### 5.1 The validation gap: beyond retrospective AUC

Most evidence for dental AI currently originates from retrospective diagnostic accuracy studies that prioritize metrics such as AUC, sensitivity, and specificity. While these metrics demonstrate technical feasibility, they are not good predictors of real-world efficacy. Randomized controlled trials and reader studies consistently demonstrate that AI assistance improves the sensitivity of practitioners, particularly junior dentists, in detecting early enamel caries (17, 85–87). These studies prove that AI can enhance diagnostic performance under controlled conditions. However, current validation studies for dental caries AI are limited by their heavy reliance on retrospective, single-center datasets, subjective expert-labeled ground truth, and diagnostic-accuracy metrics (88–91). Future studies need to:

- Move from retrospective validation to prospective, multi-center clinical trials that measure patient-centered outcomes (e.g., avoidance of unnecessary treatment, pain prevention, long-term tooth preservation) rather than only diagnostic agreement (78).
- Incorporate multi-dimensional gold standards (e.g., histology, micro-CT verification on subsets, longitudinal clinical outcomes) into trial designs to calibrate AI decision logic against biological reality.
- Evaluate decision impact and workflow ergonomics in human-in-the-loop studies; measure diagnostic synergy, decision impact on treatment trajectories, and clinician cognitive load.

### 5.2 Explainability and human–AI interaction

The black box nature of complex deep learning models remains a primary barrier to clinician trust. To address this, researchers are approaching explainability through both mathematical visualization and behavioral evaluation. Techniques such as Grad-CAM and EigenCAM have been applied to visualize model attention and provide clinicians with intuitive rationales for alerts (25, 41, 92). Eye-tracking studies have shown that in some settings, AI helps clinicians to reduce task-irrelevant attention and accelerate target locking (93). Future studies still need to:

- Standardize explainability outputs for clinical use: provide calibrated heatmaps, uncertainty estimates, and concise textual rationales that integrate with clinician workflows.
- Expand behavioral studies (eye-tracking, cognitive load measurement) to diverse clinician populations and real-world settings to ensure that explainability interventions generalize beyond laboratory conditions.
- Design interfaces that transparently present uncertainty, to mitigate automation bias and support shared decision-making with patients.

### 5.3 Integration, regulation, economics, and ethics

The viability of dental AI ultimately hinges on seamless integration into existing PACS and EHR systems, regulatory clearance, and demonstrable economic value. Regulatory pathways are maturing in major jurisdictions, and manufacturers increasingly pursue device classification and post-market surveillance (94). Economic modeling studies indicate that AI-assisted early detection can reduce lifetime treatment costs by preventing progression to complex restorative care (72, 95, 96). However, some studies show that increased detection may lead to higher treatment intensity and potential over-treatment if not contextualized within risk-based management frameworks (85).

Future studies need to:

- Prioritize integration with PACS/EHR to minimize workflow friction; evaluate integration strategies in pilot deployments.
- Plan for post-market surveillance and continual performance monitoring to detect data drift and maintain safety.
- Conduct prospective health-economic evaluations alongside clinical trials to quantify the “high initial investment, long-term payoff” profile in diverse health systems.

In addition, addressing the ethical and legal dimensions of AI usage is paramount. Regarding data privacy, the use of large-scale dental datasets requires rigorous de-identification and adherence to frameworks like GDPR or HIPAA to prevent the re-identification of patients through unique dental anatomy. Moreover, algorithmic bias must be mitigated by ensuring that training sets are representative of diverse ethnicities, socioeconomic backgrounds, and anatomical variations, thereby preventing diagnostic disparities. Meanwhile, accountability for diagnostic errors is a major legal hurdle. Current consensus suggests that AI should function as a “second reader,” and the licensed clinician is responsible for the final diagnosis and treatment. This necessitates clear professional guidelines and potentially new insurance frameworks for AI-assisted care. Finally, informed patient consent is essential; patients should be made aware when AI is utilized in their diagnosis, through transparent explanations of the system’s role and its known limitations.

## 6 Additional considerations

Some concerns and outlooks cannot be fittingly discussed solely within the data, algorithm, and translation tripartite. Here we present them as additional interrelated parts: (A) standardization and reporting, which involves a synthesis of data/algorithm/translation needs, (B) equity, access, and alignment with global health goals, which is a socio-technical concern that is beyond mere technology and involves policy and implementation considerations, and (C) large language models and genervative AI, which move beyond the traditional roles that AI assumes in dentistry.

### 6.1 Standardization and reporting

The literature shows heterogeneous reporting and inconsistent methodology, which impedes replication and meta-analysis (89, 97). The adoption of AI reporting checklists (CLAIM, TRIPOD-AI, CONSORT-AI, SPIRIT-AI, DECIDE-AI) is not yet mainstream (98–100). Journals, funders, and conferences should mandate compliance with the evolving reporting guidelines. Datasets should be published with standardized metadata (device model, acquisition parameters, patient demographics, annotation protocol, and consensus method). Benchmarking challenges should require external validation on held-out centers and report both technical metrics and clinically meaningful outcomes. These steps synthesize the data, algorithmic, and translational requirements into a coherent reproducibility and validation ecosystem.

### 6.2 Equity, access, and alignment with global health goals

Most algorithms are trained on data from well-resourced settings, which risks poor performance for underrepresented populations (88, 90, 101). Pilot deployments of mobile screening tools have shown promise for community-based screening and teledentistry (102–106). Future works should

- Intentionally collect inclusive datasets that encompass diverse ethnicities, ages, and socioeconomic backgrounds.
- Prioritize the development of lightweight, low-cost solutions deployable via mobile devices for community-based screening and teledentistry, especially in low-resource regions.
- Align AI development with public health goals (e.g., WHO targets) by focusing on early detection and prevention-oriented pathways that can be scaled in public health programs.

### 6.3 Large language models

The rapid evolution of generative AI and multimodal foundation models represents a significant shift in the dental AI landscape. Large language models and vision-language models (VLMs), such as GPT-4V, LLaVA, and Med-PaLM M, offer the potential to transcend isolated image analysis by integrating visual findings with complex clinical reasoning. Unlike traditional CNNs that output narrow classification or segmentation masks, VLMs can generate structured diagnostic reports, explain their reasoning in natural language, and answer clinician queries in real-time. In caries management, these models could theoretically synthesize radiographic evidence with patient risk factors (e.g., diet, hygiene, salivary biomarkers) to provide personalized, longitudinal treatment recommendations. However, the deployment of such models in dentistry remains in its infancy, with significant challenges regarding hallucinations in clinical contexts, high computational requirements for local deployment, and the need for domain-specific fine-tuning on high-quality dental corpora. Future research should focus on developing dental-specific VLMs that prioritize diagnostic accuracy while maintaining the interpretability required for clinical trust.

## 7 Conclusion

**Figure 1:**
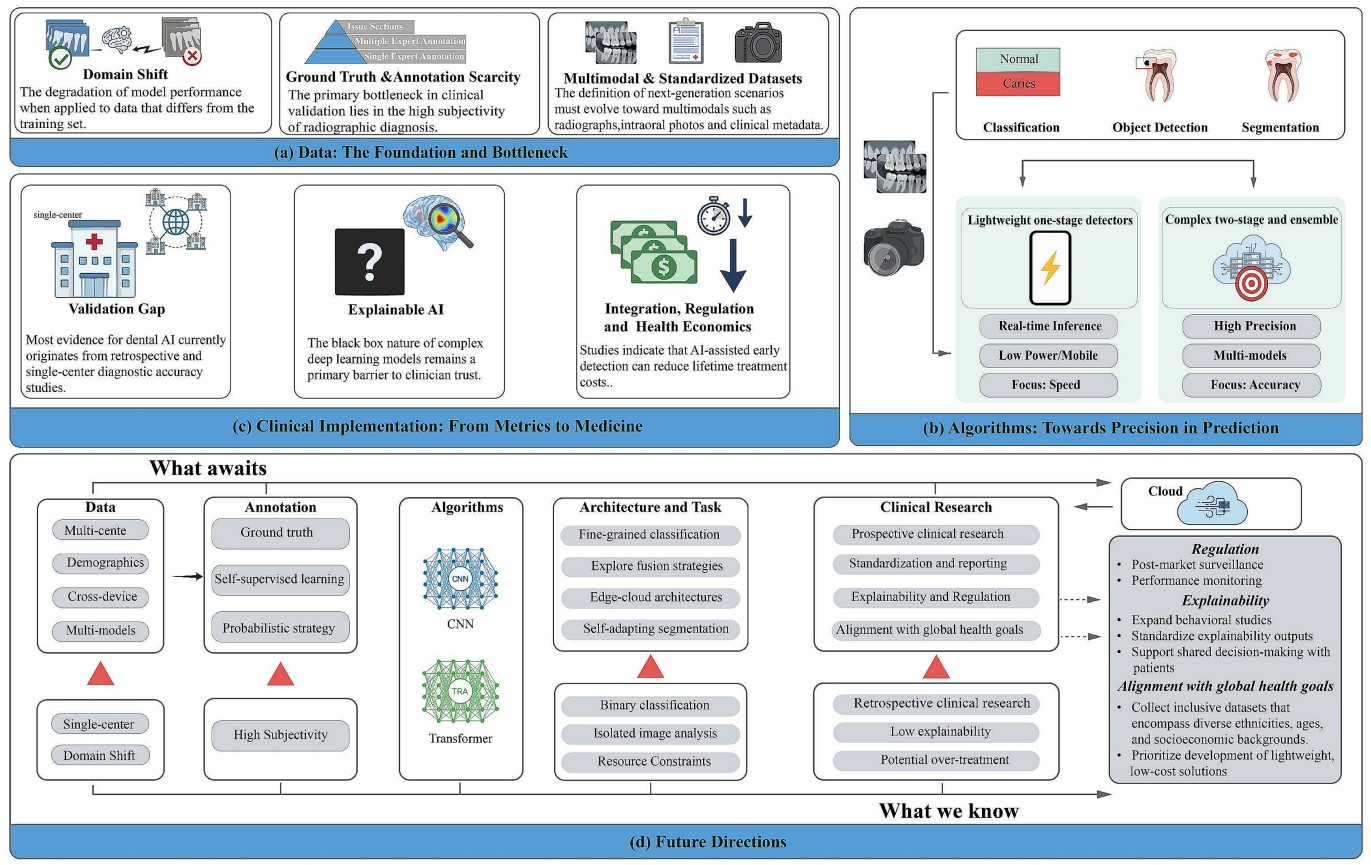
Artificial intelligence in dental caries: **(a)** Data: The foundation and bottleneck. Highlights the primary challenges in data curation, including domain shift across varying imaging conditions, the inherent subjectivity and scarcity of expert-annotated ground truth, and the necessity for standardized, multimodal datasets. **(b)** Algorithms: Toward precision in prediction. Illustrates the progression of AI tasks from binary classification to precise object detection and segmentation. It also outlines divergent deployment strategies that balance operational speed (lightweight one-stage detectors for edge inference) and diagnostic accuracy (complex ensemble models for cloud computing). **(c)** Clinical implementation: From metrics to medicine. Depicts the translational hurdles required to bridge the validation gap between in silico performance and real-world clinical utility, emphasizing the critical transition from single-center studies to robust multi-center validation. Key components also include explainable AI to foster clinician trust, alongside the integration of health economics and regulatory compliance. **(d)** Future directions. Summarizes the roadmap for next-generation caries diagnostic systems, advocating for multi-center data federation, self-supervised learning (SSL) and probabilistic annotation pipelines, hybrid architectures incorporating CNNs and Transformers, rigorous prospective clinical research, and continuous post-market surveillance.

Artificial intelligence holds transformative potential for caries diagnosis, offering a path to standardized, sensitive, and scalable detection that can address the limitations of subjective human interpretation. Technical advancements in deep learning architectures have already achieved expert-level performance on retrospective image analysis tasks. However, the journey from a high-performing algorithm to a trustworthy clinical tool is complex. It requires overcoming the fundamental challenges of data scarcity and bias, domain shift, and the subjective nature of clinical truth. Figure 1 summarizes the current landscape of AI in dental caries.

Success depends on changing the research paradigm from pursuing isolated accuracy metrics to engineering integrated systems that are evaluated through rigorous, patient-centered clinical trials. The dental AI community can ensure that the technologies fulfill their promise by embracing standardized reporting, investing in shared multimodal datasets, prioritizing explainability and human–AI collaboration, and aligning development with global oral health equity goals. Ultimately, AI cannot replace clinicians; it augments their expertise by creating a synergistic partnership that enhances early detection, promotes minimally invasive care, and reduces the global burden of a largely preventable disease.

## References

[1] Selwitz RH Ismail AI Pitts NB. Dental caries. Lancet. (2007) 369:51–9. doi: 10.1016/S0140-6736(07)60031-2, 17208642

[2] Bernabe E Marcenes W Abdulkader RS Abreu LG Afzal S Alhalaiqa FN . Trends in the global, regional, and national burden of oral conditions from 1990 to 2021: a systematic analysis for the global burden of disease study 2021. Lancet. (2025) 405:897–910. doi: 10.1016/S0140-6736(24)02811-3 40024264

[3] World Health Organization. Draft Global Oral Health Action Plan (2023–2030). Geneva: WHO (2022).

[4] Macey R Walsh T Riley P Glenny A-M Worthington HV O’Malley L . Visual or visual-tactile examination to detect and inform the diagnosis of enamel caries. Cochrane Database Syst Rev. (2021) 2021:CD014546. doi: 10.1002/14651858.CD014546 PMC8428329 34124773

[5] Stookey GK González-Cabezas C. Emerging methods of caries diagnosis. J Dent Educ. (2001) 65:1001–6. doi: 10.1002/j.0022-0337.2001.65.10.tb03441.x, 11699969

[6] Twetman S Axelsson S Dahlén G Espelid I Mejàre I Norlund A . Adjunct methods for caries detection: a systematic review of literature. Acta Odontol Scand. (2013) 71:388–97. doi: 10.3109/00016357.2012.690448, 22630355

[7] Schwendicke F Golla T Dreher M Krois J. Convolutional neural networks for dental image diagnostics: a scoping review. J Dent. (2019) 91:103226. doi: 10.1016/j.jdent.2019.103226, 31704386

[8] Qi S Fu Y Shan H Ren G Chen Y Zhang Q. Localisation and classification of multi-stage caries on CBCT images with a 3D convolutional neural network. Clin Oral Investig. (2025) 29:246. doi: 10.1007/s00784-025-06325-1, 40227550

[9] Amasya H Alkhader M Serindere G Futyma-Gąbka K Aktuna Belgin C Gusarev M . Evaluation of a decision support system developed with deep learning approach for detecting dental caries with cone-beam computed tomography imaging. Diagnostics (Basel, Switzerland). (2023) 13:3471. doi: 10.3390/diagnostics13223471, 37998607 PMC10669958

[10] Güneç HG Ürkmez EŞ Danaci A Dilmaç E Onay HH Cesur Aydin K. Comparison of artificial intelligence vs. junior dentists’ diagnostic performance based on caries and periapical infection detection on panoramic images. Quant Imaging Med Surg. (2023) 13:7494–503. doi: 10.21037/qims-23-762, 37969638 PMC10644137

[11] Tichý A Kunt L Nagyová V Kybic J. Automatic caries detection in bitewing radiographs—part II: experimental comparison. Clin Oral Investig. (2024) 28:133. doi: 10.1007/s00784-024-05528-2, 38315246 PMC10844156

[12] Zhu J Chen Z Zhao J Yu Y Li X Shi K . Artificial intelligence in the diagnosis of dental diseases on panoramic radiographs: a preliminary study. BMC Oral Health. (2023) 23:358. doi: 10.1186/s12903-023-03027-6, 37270488 PMC10239110

[13] Tirkkonen O Tiensuu H Väyrynen E Suutala J Vuollo V Laitala M-L . An explainable and transparent machine learning approach for predicting dental caries: a cross-national validation study. BMC Oral Health. (2026) 26:280. doi: 10.1186/s12903-026-07660-9, 41546040 PMC12896046

[14] Sastika B Marappan R. AI-driven approaches for enhancing early detection of dental caries. 2025 International Conference on Intelligent Computing and Knowledge Extraction (ICICKE). (2025). p. 1–5

[15] Rodrigues N Martinez-Rus F Miguel-Calvo A Pradíes G Salido MP. Accuracy assessment of human and artificial intelligence-assisted bitewing radiography and near-infrared reflectance imaging-based methods for interproximal caries detection: a histological validation. Caries Res. (2025) 60:173–84. doi: 10.1159/000546644, 40451163

[16] Kunt L Kybic J Nagyová V Tichý A. Automatic caries detection in bitewing radiographs: part I—deep learning. Clin Oral Investig. (2023) 27:7463–71. doi: 10.1007/s00784-023-05335-1, 37968358

[17] Luke AM Rezallah NNF. Accuracy of artificial intelligence in caries detection: a systematic review and meta-analysis. Head Face Med. (2025) 21:24. doi: 10.1186/s13005-025-00496-8, 40181403 PMC11969992

[18] Nagareddy B Vadlamani R Venkannagari NR Jain S Basheer SN Murugesan S. Comparison of the artificial intelligence versus traditional radiographic interpretation in detecting periapical periodontitis: a diagnostic accuracy study. J Pharm Bioallied Sci. (2024) 16:S3676–8. doi: 10.4103/jpbs.jpbs_1096_24, 39926764 PMC11805257

[19] Zhang J-W Fan J Zhao F-B Ma B Shen X-Q Geng Y-M. Diagnostic accuracy of artificial intelligence-assisted caries detection: a clinical evaluation. BMC Oral Health. (2024) 24:1095. doi: 10.1186/s12903-024-04847-w, 39285427 PMC11406783

[20] Schwarzmaier J Frenkel E Neumayr J Ammar N Kessler A Schwendicke F . Validation of an artificial intelligence-based model for early childhood caries detection in dental photographs. J Clin Med. (2024) 13:13. doi: 10.3390/jcm13175215, 39274428 PMC11396146

[21] van Nistelrooij N Chaves ET Cenci MS Cao L Loomans BAC Xi T . Deep learning-based algorithm for staging secondary caries in bitewings. Caries Res. (2025) 59:163–73. doi: 10.1159/000542289, 39471790 PMC12263131

[22] Kwiatek J Leśna M Piskórz W Kaczewiak J. Comparison of the diagnostic accuracy of an AI-based system for dental caries detection and clinical evaluation conducted by dentists. J Clin Med. (2025) 14:14. doi: 10.3390/jcm14051566, 40095536 PMC11900972

[23] Tareq A Faisal MI Islam MS Rafa NS Chowdhury T Ahmed S . Visual diagnostics of dental caries through deep learning of non-standardised photographs using a hybrid YOLO ensemble and transfer learning model. Int J Environ Res Public Health. (2023) 20:20. doi: 10.3390/ijerph20075351, 37047966 PMC10094335

[24] Inani H Mehta V Bhavsar D Gupta RK Jain A Akhtar Z. AI-enabled dental caries detection using transfer learning and gradient-based class activation mapping. J Ambient Intell Humaniz Comput. (2024) 15:3009–33. doi: 10.1007/s12652-024-04795-x

[25] Kim D Kim J Choi SG. CNN-based remote dental diagnosis model for caries detection with grad-CAM. Sci Rep. (2025) 15:26555. doi: 10.1038/s41598-025-11447-3, 40695984 PMC12283948

[26] Kim MJ Chae SG Bae SJ Hwang K-G. Unsupervised few shot learning architecture for diagnosis of periodontal disease in dental panoramic radiographs. Sci Rep. (2024) 14:23237. doi: 10.1038/s41598-024-73665-5, 39369017 PMC11455883

[27] Zanini LGK Rubira-Bullen IRF Nunes F d LDS. Enhancing dental caries classification in CBCT images by using image processing and self-supervised learning. Comput Biol Med. (2024) 183:109221. doi: 10.1016/j.compbiomed.2024.109221, 39378579

[28] Almalki A Almalki A Jan Latecki L. Self-supervised masked deep embeddings for dental caries detection. IEEE Access. (2025) 13:161206–16. doi: 10.1109/ACCESS.2025.3606811

[29] Vijayarajan V Sindha MMR Pandyan UM. "Comparative evaluation of image enhancement techniques for enhanced tissue visualization in dental panoramic radiographs". In: Sathees Kumaran S Sridevi S Zhang Y-D Emilia Balas V Hong T-P, editors. Innovative and Intelligent Computing for Global Sustainability. Cham: Springer Nature Switzerland (2026). p. 205–14.

[30] Waite S Scott J Gale B Fuchs T Kolla S Reede D. Interpretive error in radiology. Am J Roentgenol. (2017) 208:739–49. doi: 10.2214/AJR.16.16963, 28026210

[31] Chen P-HC Mermel CH Liu Y. Evaluation of artificial intelligence on a reference standard based on subjective interpretation. Lancet Digit Health. (2021) 3:e693–5. doi: 10.1016/S2589-7500(21)00216-8, 34561202

[32] Pérez de Frutos J Holden Helland R Desai S Nymoen LC Langø T Remman T . AI-Dentify: deep learning for proximal caries detection on bitewing x-ray - HUNT4 Oral health study. BMC Oral Health. (2024) 24:344. doi: 10.1186/s12903-024-04120-0, 38494481 PMC10946166

[33] Hu M Zhang Q Wei Z Jia P Yuan M Yu H . Self-supervised learning enhances periapical films segmentation with limited labeled data. J Dent. (2025) 163:106150. doi: 10.1016/j.jdent.2025.106150, 41067573

[34] Wang C Zhang Y Wu C Liu J Huang X Wu L . MMDental - a multimodal dataset of tooth CBCT images with expert medical records. Sci Data. (2025) 12:1172. doi: 10.1038/s41597-025-05398-7, 40634413 PMC12241571

[35] Ngnamsie Njimbouom S Lee K Kim J-D. MMDCP: multi-modal dental caries prediction for decision support system using deep learning. Int J Environ Res Public Health. (2022) 19:10928. doi: 10.3390/ijerph191710928, 36078635 PMC9518085

[36] Schneider H Ahrens M Strumpski M Rüger C Häfer M Hüttmann G . An intraoral OCT probe to enhanced detection of Approximal carious lesions and assessment of restorations. J Clin Med. (2020) 9:9. doi: 10.3390/jcm9103257, 33053724 PMC7600310

[37] Braga AS Meißner T Schulz-Kornas E Haak R Magalhães AC Esteves-Oliveira M. Enamel caries lesion depth obtained by optical coherence tomography and transverse microradiography: a comparative study. Caries Res. (2024) 58:502–10. doi: 10.1159/000539406, 38763130

[38] Esmaeilyfard R Bonyadifard H Paknahad M. Dental caries detection and classification in CBCT images using deep learning. Int Dent J. (2024) 74:328–34. doi: 10.1016/j.identj.2023.10.003, 37940474 PMC10988262

[39] Heo M-S. Caries is a gradient, not a boundary: detection rather than segmentation is the appropriate deep learning approach. Imaging Sci Dent. (2025) 55:319–21. doi: 10.5624/isd.20251201, 41536895 PMC12798388

[40] Lian L Zhu T Zhu F Zhu H. Deep learning for caries detection and classification. Diagnostics. (2021) 11:1672. doi: 10.3390/diagnostics11091672, 34574013 PMC8469830

[41] Oztekin F Katar O Sadak F Yildirim M Cakar H Aydogan M . An explainable deep learning model to prediction dental caries using panoramic radiograph images. Diagnostics. (2023) 13:226. doi: 10.3390/diagnostics13020226, 36673036 PMC9858273

[42] Schwendicke F Cejudo Grano de Oro J Garcia Cantu A Meyer-Lueckel H Chaurasia A Krois J. Artificial intelligence for caries detection: value of data and information. J Dent Res. (2022) 101:1350–6. doi: 10.1177/00220345221113756, 35996332 PMC9516598

[43] Zhou X Yu G Yin Q Liu Y Zhang Z Sun J. Context aware convolutional neural network for children caries diagnosis on dental panoramic radiographs. Comput Math Methods Med. (2022) 2022:1–8. doi: 10.1155/2022/6029245, 36188109 PMC9519291

[44] Liu Y Xia K Cen Y Ying S Zhao Z. Artificial intelligence for caries detection: a novel diagnostic tool using deep learning algorithms. Oral Radiol. (2024) 40:375–84. doi: 10.1007/s11282-024-00741-x, 38498223

[45] Jeon KJ Ha E-G Choi H Lee C Han S-S. Performance comparison of three deep learning models for impacted mesiodens detection on periapical radiographs. Sci Rep. (2022) 12:15402. doi: 10.1038/s41598-022-19753-w, 36100696 PMC9470664

[46] Fatima S Haider NG Riaz R. YOLOv8 vs RetinaNet vs EfficientDet: a comparative analysis for modern object detection. Int J Emerg Eng Technol. (2024) 3:1–5. doi: 10.57041/3j4psw71

[47] Salehizeinabadi M Neghab S Ameli N Baghi KK Pacheco-Pereira C. Automated classification of dental caries in bitewing radiographs using machine learning and the ICCMS framework. Int J Dent. (2025) 2025:6644310. doi: 10.1155/ijod/6644310, 40894183 PMC12393923

[48] Karakuş R Öziç MÜ Tassoker M. AI-assisted detection of interproximal, occlusal, and secondary caries on bite-wing radiographs: a single-shot deep learning approach. J Imaging Inform Med. (2024) 37:3146–59. doi: 10.1007/s10278-024-01113-x, 38743125 PMC11612078

[49] Faizan Ahmed SM Ghori MH Khalid A Nooruddin A Adnan N Lal A . Annotated intraoral image dataset for dental caries detection. Sci Data. (2025) 12:1297. doi: 10.1038/s41597-025-05647-9 40715095 PMC12297690

[50] Alsolamy M Nadeem F Azhari AA Ahmed WM. Automated detection, localization, and severity assessment of proximal dental caries from bitewing radiographs using deep learning. Diagnostics. (2025) 15:899. doi: 10.3390/diagnostics15070899, 40218248 PMC11988774

[51] Zhou W Zhao D Zhao B Shen M Liu X. A deep learning framework for automated dental segmentation and diagnostic report generation from cone-beam computed tomography. Head Face Med. (2025) 21:80. doi: 10.1186/s13005-025-00555-0, 41316386 PMC12661820

[52] Kot WY Au Yeung SY Leung YY Leung PH Yang W. Evolution of deep learning tooth segmentation from CT/CBCT images: a systematic review and meta-analysis. BMC Oral Health. (2025) 25:800. doi: 10.1186/s12903-025-05984-6, 40420051 PMC12107724

[53] Ji C Liu Y He L Jiang Y Huang C Wang L. "A two-stage semi-supervised nnU-net model for automated tooth segmentation in panoramic X-ray images". In: Wang Y Qian D Wang S Ben-Hamadou A Pujades S Lumetti L , editors. Supervised and Semi-Supervised Multi-Structure Segmentation and Landmark Detection in Dental Data. Cham: Springer Nature Switzerland (2025). p. 91–9.

[54] Liu J Zhang H Chen J Meng R Gao C Han L . Automated detection and segmentation of dental caries using a novel cascaded learning approach. Biomed Signal Process Control. (2025) 102:107344. doi: 10.1016/j.bspc.2024.107344

[55] Jones B Lambach M Chen T Michou S Kilpatrick N Curtis N . Dental caries detection in children using intraoral scans and deep learning. J Dent. (2025) 160:105906. doi: 10.1016/j.jdent.2025.105906, 40527440

[56] Tez BÇ Güzel Y Kızıltan Eliaçık BB Aydın Z. Deep-learning-based AI-model for predicting dental plaque in the young permanent teeth of children aged 8–13 years. Children (Basel). (2025) 12:475. doi: 10.3390/children12040475 40310101 PMC12025585

[57] Schneider L Krasowski A Pitchika V Bombeck L Schwendicke F Büttner M. Assessment of CNNs, transformers, and hybrid architectures in dental image segmentation. J Dent. (2025) 156:105668. doi: 10.1016/j.jdent.2025.105668, 40064460

[58] Lee S Oh S Jo J Kang S Shin Y Park J. Deep learning for early dental caries detection in bitewing radiographs. Sci Rep. (2021) 11:16807. doi: 10.1038/s41598-021-96368-7, 34413414 PMC8376948

[59] Ragab MG Abdulkadir SJ Muneer A Alqushaibi A Sumiea EH Qureshi R . A comprehensive systematic review of YOLO for medical object detection (2018 to 2023). IEEE Access. (2018) 12:57815–36. doi: 10.1109/ACCESS.2024.3386826

[60] Zhou Y. A YOLO-NL object detector for real-time detection. Expert Syst Appl. (2024) 238:122256. doi: 10.1016/j.eswa.2023.122256

[61] Ji S-J Ling Q-H Han F. An improved algorithm for small object detection based on YOLO v4 and multi-scale contextual information. Comput Electr Eng. (2023) 105:108490. doi: 10.1016/j.compeleceng.2022.108490

[62] Craioveanu M Stamatescu G Popescu D. Ensemble strategy with multi-step hard sample mining for improved UXO localisation and classification. IEEE Access. (2025) 13:123546–58. doi: 10.1109/ACCESS.2025.3588184

[63] Casado-García Á Heras J. Ensemble methods for object detection. ECAI 2020. IOS Press (2020). p. 2688–2695

[64] Xu J Wang W Wang H Guo J. Multi-model ensemble with rich spatial information for object detection. Pattern Recogn. (2020) 99:107098. doi: 10.1016/j.patcog.2019.107098

[65] Felsch M Meyer O Schlickenrieder A Engels P Schönewolf J Zöllner F . Detection and localization of caries and hypomineralization on dental photographs with a vision transformer model. NPJ Digit Med. (2023) 6:198. doi: 10.1038/s41746-023-00944-2, 37880375 PMC10600213

[66] Balhaddad AA Alharamlah F Albrahim HF Ahmad S Melo MAS Mokeem L . Assessing diagnostic accuracy and monitoring of caries progression using optical coherence tomography (OCT): a systematic review. J Dent. (2025) 155:105628. doi: 10.1016/j.jdent.2025.105628, 39954804

[67] Strumpski M Schneider H Rüger C Schmidt J Schulz-Kornas E Haak R. Validity and reliability of intraoral optical coherence tomography and bitewing radiography for detecting approximal carious lesions. Caries Res. (2025) 59:476–89. doi: 10.1159/000544789, 39999820 PMC12688330

[68] Tashkandi AK Jiffri SO Albalawi RM Albukhari SA Mugharbil SA Yeslam HE. Detection of proximal dental caries in primary teeth with a near-infrared-irradiation-assisted intraoral scanner: an in vitro study. BMC Oral Health. (2025) 25:270. doi: 10.1186/s12903-025-05629-8, 39979907 PMC11844066

[69] Hsieh S-T Cheng Y-A. Multimodal feature fusion in deep learning for comprehensive dental condition classification. J Xray Sci Technol. (2024) 32:303–21. doi: 10.3233/XST-230271, 38217632

[70] Bui TH Hamamoto K Paing MP. Deep fusion feature extraction for caries detection on dental panoramic radiographs. Appl Sci. (2021) 11:2005. doi: 10.3390/app11052005, 30654563

[71] Wang C Xu H Tang H Xin L Huang X Wang N . Fluorescence and reflectance-based dual-modal hyperspectral image fusion for caries diagnosis. Measurement. (2025) 246:116701. doi: 10.1016/j.measurement.2025.116701

[72] Jones B Chen T Michou S Kilpatrick N Curtis N Burgner DP . Dental caries detection in children using intraoral scanners featuring fluorescence: diagnostic agreement study. JMIR Public Health Surveill. (2025) 11:e78023. doi: 10.2196/78023, 41358644 PMC12683709

[73] Salahin SMS Ullaa MDS Ahmed S Mohammed N Farook TH Dudley J. One-stage methods of computer vision object detection to classify carious lesions from smartphone imaging. Oral. (2023) 3:176–90. doi: 10.3390/oral3020016

[74] Thanh MTG Toan NV Ngoc VTN Tra NT Giap CN Nguyen DM. Deep learning application in dental caries detection using intraoral photos taken by smartphones. Appl Sci. (2022) 12:5504. doi: 10.3390/app12115504

[75] Yang L Chen G-Y. Evaluation of deep learning for caries detection with fine-grained classification and postprocessing improvements. Int Dent J. (2025) 75:100898. doi: 10.1016/j.identj.2025.100898, 40701111 PMC12305711

[76] Huy H-Q Ngoc N-C Tuan N-T. Two-stage framework for automated caries segmentation: from ROI-based prediction to full-image mask reconstruction. 2025 International Symposium on Electrical and Electronics Engineering (ISEE). (2025). p. 30–35

[77] Zhang S Cui Y Xu D Lin Y. A collaborative inference strategy for medical image diagnosis in mobile edge computing environment. PeerJ Comput Sci. (2025) 11:e2708. doi: 10.7717/peerj-cs.2708, 40134868 PMC11935760

[78] Balachandran S Marappan R. EnamelNet-TRiX: a lesion-aware dual-transformer with cross-attention for early and advanced enamel caries diagnosis. Int Dent J. (2026) 76:109437. doi: 10.1016/j.identj.2026.109437, 41950800 PMC13090983

[79] Jameil AK Al-Raweshidy H. A digital twin framework for real-time healthcare monitoring: leveraging AI and secure systems for enhanced patient outcomes. Discov Internet Things. (2025) 5:37. doi: 10.1007/s43926-025-00135-3

[80] Jameil AK Al-Raweshidy H. Hybrid cloud-edge AI framework for real-time predictive analytics in digital twin healthcare systems (2024). doi: 10.21203/rs.3.rs-5412158/v1

[81] Weber T Dexl J Rügamer D Ingrisch M. Post-training network compression for 3D medical image segmentation: reducing computational efforts via Tucker decomposition. Radiol Artif Intell. (2025) 7:e240353. doi: 10.1148/ryai.240353 39812583

[82] Qi Y Zhang W Wang X You X Hu S Chen J. Efficient knowledge distillation for brain tumor segmentation. Appl Sci. (2022) 12:12. doi: 10.3390/app122311980, 30654563

[83] Isensee F Jaeger PF Kohl SAA Petersen J Maier-Hein KH. nnU-net: a self-configuring method for deep learning-based biomedical image segmentation. Nat Methods. (2021) 18:203–11. doi: 10.1038/s41592-020-01008-z, 33288961

[84] Greenhalgh T Wherton J Papoutsi C Lynch J Hughes G A’Court C . Beyond adoption: a new framework for theorizing and evaluating nonadoption, abandonment, and challenges to the scale-up, spread, and sustainability of health and care technologies. J Med Internet Res. (2017) 19:e367. doi: 10.2196/jmir.8775 29092808 PMC5688245

[85] Mertens S Krois J Cantu AG Arsiwala LT Schwendicke F. Artificial intelligence for caries detection: randomized trial. J Dent. (2021) 115:103849. doi: 10.1016/j.jdent.2021.103849, 34656656

[86] Adnan N Faizan Ahmed SM Das JK Aijaz S Sukhia RH Hoodbhoy Z . Developing an AI-based application for caries index detection on intraoral photographs. Sci Rep. (2024) 14:26752. doi: 10.1038/s41598-024-78184-x, 39500993 PMC11538444

[87] Ammar N Kühnisch J. Diagnostic performance of artificial intelligence-aided caries detection on bitewing radiographs: a systematic review and meta-analysis. Jpn Dent Sci Rev. (2024) 60:128–36. doi: 10.1016/j.jdsr.2024.02.001, 38450159 PMC10917640

[88] Albano D Galiano V Basile M Di Luca F Gitto S Messina C . Artificial intelligence for radiographic imaging detection of caries lesions: a systematic review. BMC Oral Health. (2024) 24:274. doi: 10.1186/s12903-024-04046-7, 38402191 PMC10894487

[89] Mohammad-Rahimi H Motamedian SR Rohban MH Krois J Uribe SE Mahmoudinia E . Deep learning for caries detection: a systematic review. J Dent. (2022) 122:104115. doi: 10.1016/j.jdent.2022.104115, 35367318

[90] Hung M Yevseyevich D Khazana M Schwartz C Lipsky MS. Charting new territory: AI applications in dental caries detection from panoramic imaging. Dent J. (2025) 13:13. doi: 10.3390/dj13080366, 40863069 PMC12385533

[91] Moharrami M Farmer J Singhal S Watson E Glogauer M Johnson AEW . Detecting dental caries on oral photographs using artificial intelligence: a systematic review. Oral Dis. (2024) 30:1765–83. doi: 10.1111/odi.14659, 37392423

[92] Qi S Shan H Fu Y Chen Y Zhang Q. A clinically oriented and interpretable AI framework for classifying dentin caries severity on CBCT images. J Prosthet Dent. (2025) 135:594.e1–8. doi: 10.1016/j.prosdent.2025.10.034, 41177738

[93] Arsiwala-Scheppach LT Castner NJ Rohrer C Mertens S Kasneci E Cejudo Grano de Oro JE . Impact of artificial intelligence on dentists’ gaze during caries detection: a randomized controlled trial. J Dent. (2024) 140:104793. doi: 10.1016/j.jdent.2023.104793, 38016620

[94] Shujaat S Aljadaan H Alrashid H Aboalela AA Riaz M. FDA-approved AI solutions in dental imaging: a narrative review of applications, evidence, and outlook. Int Dent J. (2025) 76:109315. doi: 10.1016/j.identj.2025.109315, 41421004 PMC12775797

[95] Schwendicke F Rossi JG Göstemeyer G Elhennawy K Cantu AG Gaudin R . Cost-effectiveness of artificial intelligence for proximal caries detection. J Dent Res. (2021) 100:369–76. doi: 10.1177/0022034520972335, 33198554 PMC7985854

[96] Gomez Rossi J Rojas-Perilla N Krois J Schwendicke F. Cost-effectiveness of artificial intelligence as a decision-support system applied to the detection and grading of melanoma, dental caries, and diabetic retinopathy. JAMA Netw Open. (2022) 5:e220269. doi: 10.1001/jamanetworkopen.2022.0269, 35289862 PMC8924723

[97] Maiter A. Quality of reporting of studies on artificial intelligence in radiology: what is the current state of the field? Diagn Interv Radiol. (2025). doi: 10.4274/dir.2025.253315, 40321088 PMC13530544

[98] Ndiaye AD Gasqui MA Millioz F Perard M Leye Benoist F Grosgogeat B. Exploring the methodological approaches of studies on radiographic databases used in Cariology to feed artificial intelligence: a systematic review. Caries Res. (2024) 58:117–40. doi: 10.1159/000536277, 38342096

[99] Liu X Cruz Rivera S Moher D Calvert MJ Denniston AK SPIRIT-AI and CONSORT-AI Working Group. Reporting guidelines for clinical trial reports for interventions involving artificial intelligence: the CONSORT-AI extension. Nat Med. (2020) 26:1364–74. doi: 10.1038/s41591-020-1034-x 32908283 PMC7598943

[100] Mongan J Moy L Kahn CE. Checklist for artificial intelligence in medical imaging (CLAIM): a guide for authors and reviewers. Radiol Artif Intell. (2020) 2:e200029. doi: 10.1148/ryai.2020200029, 33937821 PMC8017414

[101] Sande A Mathur A Sapkal R Tamboli A. Applications of AI-based deep learning models for detecting dental caries on intraoral images – a systematic review. J Neonatal Surg. (2025) 14:523–33. doi: 10.52783/jns.v14.1827 39609513

[102] Estai M Kanagasingam Y Huang B Shiikha J Kruger E Bunt S . Comparison of a smartphone-based photographic method with face-to-face caries assessment: a mobile teledentistry model. Telemed J E Health. (2017) 23:435–40. doi: 10.1089/tmj.2016.0122, 27854186

[103] Qari AH Hadi M Alaidarous A Aboalreesh A Alqahtani M Bamaga IK . The accuracy of asynchronous tele-screening for detecting dental caries in patient-captured mobile photos: a pilot study. Saudi Dent J. (2024) 36:105–11. doi: 10.1016/j.sdentj.2023.10.006, 38375381 PMC10874790

[104] Sharkawy MN Mohamed M Abbas HM. Accuracy of teledentistry versus clinical oral examination for aged-care home residents: a pilot study. J Frailty Aging. (2025) 14:100001. doi: 10.1016/j.tjfa.2024.100001, 39855885 PMC12184020

[105] Iranparvar P Ghorbani Z Zandieh S Hartoonian S Shirazi M. Evaluating maternal accuracy in smartphone-based tele-dentistry for early childhood caries detection. Clin Exp Dent Res. (2025) 11:e70231. doi: 10.1002/cre2.70231, 41039962 PMC12491845

[106] Lamas-Lara VF Mattos-Vela MA Evaristo-Chiyong TA Guerrero ME Jiménez-Yano JF Gómez-Meza DN. Validity and reliability of a smartphone-based photographic method for detection of dental caries in adults for use in teledentistry. Front Oral Health. (2025) 6:1470706. doi: 10.3389/froh.2025.1470706, 40443702 PMC12119532

